# Sagittal and Frontal Plane Evaluation of the Whole Spine and Clinical Outcomes after Vertebral Fractures

**DOI:** 10.1155/2015/787904

**Published:** 2015-10-08

**Authors:** A. Topalidou, G. Tzagarakis, K. Balalis, K. Ziogas, A. Papaioannou

**Affiliations:** ^1^Faculty of Medicine, Department of Orthopaedics and Traumatology, University Hospital of Heraklion, University of Crete, 71003 Heraklion, Greece; ^2^Faculty of Medicine, Department of Anaesthesiology, University Hospital of Heraklion, University of Crete, 71003 Heraklion, Greece

## Abstract

Although it is known that a change in any level of the spine alters biomechanics, there are not many studies to evaluate the spine as a whole in both sagittal and frontal planes. This prospective cohort study evaluates the morphology and mobility of the entire spine in patients with vertebral fractures. The Treatment Group consisted of 43 patients who underwent percutaneous balloon kyphoplasty or percutaneous balloon kyphoplasty plus fixation. The Control Group consisted of 39 healthy subjects. Spinal Mouse was used for the assessment of the curvatures and the mobility of the spine. Clinical outcomes were evaluated by Visual Analogue Scale and Oswestry Disability Index. The measurements were recorded at 15 days and 3, 6, and 12 months postoperatively. Regarding the curvatures and mobility in sagittal plane, a statistically significant increase appeared early at 3 months, for lumbar curve, spinopelvic angulation, and overall trunk inclination. In the frontal plane, most of the improvements were recorded after 6 months. Patients with osteoporotic fracture showed statistically significant lower mean value than patients with traumatic fracture. Pain and disability index showed early improvements. This study provides a comprehensive and complete picture of the functionality of the spine in patients treated with percutaneous balloon kyphoplasty.

## 1. Introduction

It is estimated that every year over 1.4 million people worldwide sustain vertebral fractures (VFs) [1], mainly due to osteoporosis and secondly due to other causes such as trauma, neoplasm, and infection [2, 3]. A VF, apart from pain and in some cases neurologic deficit, may result in functional impairment, gradual curvature deformity, abnormal posture, decreased mobility, and balance distortion [4–6]. In addition, it is stated that all the above and mainly the disturbance of the spinal mobility have a negative effect in the quality of life (QOL) in these patients [7, 8].

Treatment of VFs includes percutaneous balloon kyphoplasty (BKP) and BKP plus fixation [4, 7, 9, 10]. The postoperative outcome, in most of the studies, is assessed with regard either to the thoracic curvature (kyphosis) or to the lumbar lordosis. However, it is known that the changes in the morphology and the mobility of the spine affect the global spine. Moreover, most of the studies do not investigate the spinopelvic angulation and the hip sacral mobility, even though it has been proven that when there is a spine deformity this angle changes as a compensatory mechanism and thus influence the balance of the patient [6–8, 11]. Many methods have been used for the assessment of the spine. Nevertheless, most of them either have a poor reliability and poor validity and are time-consuming [12–14] or contain the risk of radiation [15]. To the best of our knowledge no studies have investigated the spine as a whole. There is no reference in the literature examining the frontal plane in people with osteoporosis or in people with VF.

For the abovementioned reasons, the purpose of the present study is to provide further evidence for the evaluation of the morphology and functionality of the global spine, in patients with VF, with a new valid, reliable, and noninvasive method both in sagittal and in frontal planes.

## 2. Materials and Methods

### 2.1. Subjects

From September 2010 to December 2012, 43 patients were treated (Treatment Group, TG) with BKP or BKP plus short minimal invasive fixation, due to osteoporotic or traumatic VF in the thoracic, lumbar, or thoracolumbar spine. All patients were followed up for one year postoperatively. Thirty-nine completed the full evaluation protocol. Two of the patients presented fracture in an inferior level between 6 and 12 months and two abandoned our study for personal reasons. The diagnosis of VF was made by plain radiography, Computed Tomography (CT), and/or Magnetic Resonance Imaging (MRI). All patients' profiles were assessed regarding the appropriateness for kyphoplasty procedure. Exclusion criteria were previous vertebroplasty or balloon kyphoplasty or other spine surgeries, pedicle fractures, local or systemic infection, preexisting chronic back pain or inability to stand, hemiplegia or stroke, ankylosing spondylitis, spondyloarthropathy, dementia, psychiatric history or other mental inabilities to participate in the study, and age higher than 75 years. All subjects were operated on by the same orthopaedic surgeon at the same center.

Thirty-nine healthy subjects who had no pathology of the spine or the lower limbs comprised the Control Group (CG). All of them had no history of neuromuscular and musculoskeletal pathology or injury.

All participants were informed in detail on the purpose of the study and signed an informed consent form approved by the Bioethics and Scientific Committee of the University Hospital of Heraklion (10787/20-12-10).

### 2.2. Technique

**Figure 1:**
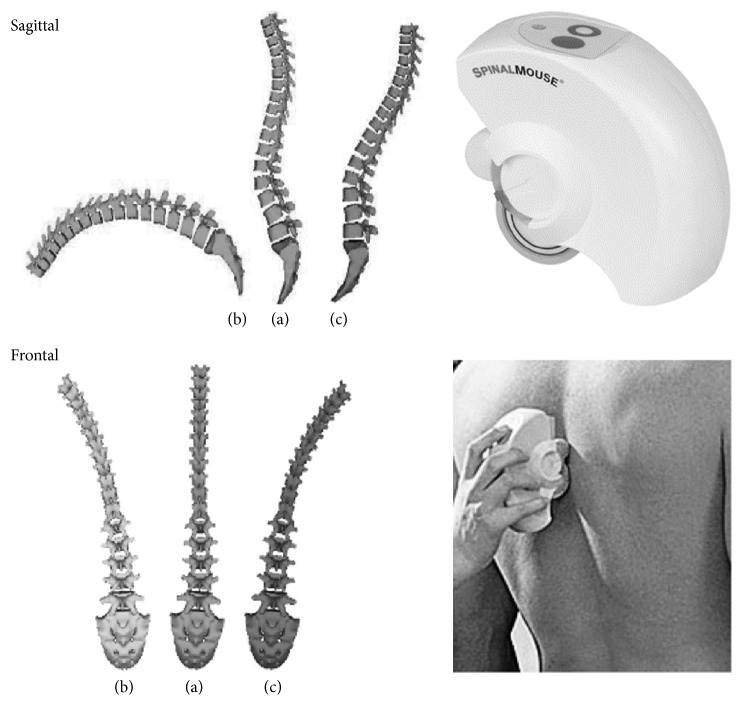
Spinal Mouse is a device which is guided manually on the skin along the spine. Reconstruction of the spine in neutral and extreme positions in sagittal and frontal plane. The images are derived from real measurements in one patient. Sagittal plane: (a) upright position, (b) full flexion, and (c) full extension. Frontal level: (a) upright position, (b) left lateral bending, and (c) right lateral bending.

Regarding the evaluation of the spine, both groups were assessed with Spinal Mouse (Idiag, Volketswil, Switzerland), a computer-assisted wireless telemetry device, which is guided along the spinous processes of the vertebral column. A computer device receives all the data obtained by the Spinal Mouse in real time and reproduces a two-dimensional graph of the spine (Figure 1). The recording frequency was 150 Hz. A periodical algorithm was used for the calculation of the mobility of the curves.

Only the subjects of the TG were asked to fill two questionnaires. Back pain was evaluated using the Visual Analogue Scale (VAS: 0 = no pain at all, 10 = worst pain imaginable) [16]. The functional disability was evaluated using the Oswestry Disability Index (ODI: 0 = minimal to 100% = maximal disability) [17]. Follow-up measurements and questionnaires completion were performed in 15 days and 3, 6, and 12 months postoperatively (±one calendar week). There was no possibility for preoperative measurements, because most of the participants of TG group were bedridden and had pain.

CG spinal function and mobility were evaluated at the same environment with TG. Subjects in CG were assessed only once.

The same procedure and order were followed for all measurements. This particular measurement technique and the parameters which were counted have been described in the literature [18].

### 2.3. Statistical Analysis

Paired *t*-test and repeated measures analysis of variance (ANOVA) were used to test whether there was a significant surgery effect on Spinal Mouse's parameters at 15 days and 3, 6, and 12 months postoperatively. In the case of a statistically significant finding, post hoc Bonferroni adjusted tests were needed to pinpoint differences. 95% confidence interval (CI) was also computed in order to obtain a clearer estimation of these parameters. ANOVA and post hoc Bonferroni adjustment were used to test the influence of surgery in ODI and VAS in all reevaluations.

One-way ANOVA was used to determine whether there were any significant differences between the means of CG, in comparison with the mean values of TG of the 12-month postoperative reevaluation. It was also used to compare the means of patients that were treated with BKP and those who were treated by using BKP plus short minimal invasive fixation and to compare the means of groups' patients based on type of fracture (osteoporotic and traumatic).

SPPS 15.0 was used for statistical analysis. All statistical tests were carried at the 5% level of significance.

## 3. Results

**Figure 2:**
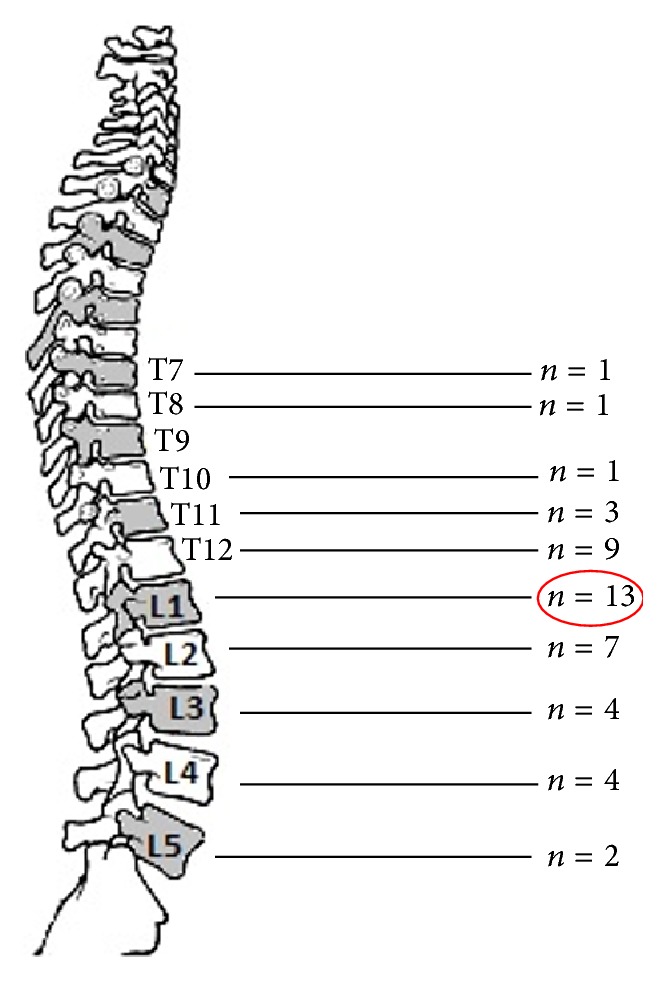
The total number of fractures that appeared in each level. L1 showed the greatest possibility for fracture (28.9%).

Demographic and anthropometric characteristics of TG and CG are presented in Table 1. Twenty-three patients from TG were operated on due to osteoporotic vertebral fractures and 16 due to traumatic fractures. In total, 45 fractured vertebrae were treated. The number of the vertebrae that had fracture in every level is shown in Figure 2. Thirty-one patients were treated with BKP and the remaining 8 with BKP plus short minimal invasive fixation.

### 3.1. Spine Curvatures

#### 3.1.1. Sagittal Plane

The statistically significant changes are mainly presented in the reevaluations of 3, 6, and 12 months, in comparison with the measurement in 15 days postoperatively. Improvement in the thoracic curvature appears only during the measurement in the position of full extension. Statistically significant increase for the lumbar curve appears early at 3 months, in comparison with the 15-day evaluation, which in upright position was maintained up to 12 months, while in full flexion and full extension it continues to show a slight increase up to 12 months. It is worth mentioning that, in the upright position, lumbar curve was 17.85° in 15 days, increased to 23.7° in the 3-month evaluation, and remained almost unchanged up to 12 months. Finally, statistically significant improvements are shown in spinopelvic angulation (hip sacral angle, Sac_Hip) and in the overall trunk inclination (Incl).

#### 3.1.2. Frontal Plane

There were no statistically significant changes in the upright position regarding lumbar and thoracic curvatures. Statistically significant improvements for right and left lateral bending positions for the thoracic curve were observed at the 6-month evaluation, but for the lumbar curve in 12 months compared with the 15-day reevaluation.

The statistically significant changes that were recorded in the sagittal and frontal planes are presented in Table 2.

### 3.2. Spine Mobility

#### 3.2.1. Sagittal Plane

Few of the parameters showed statistically significant differences, between the 3- and 6-month measurements. Most of the parameters exhibited improvement already from 3 months. A typical example is the increase of range of motion (ROM) of lumbar curvature from the upright position to full flexion (AF). In 15 days it was 7.9° ± 3.04° and in 3 months 41.08° ± 2.95°. Other parameters increased significantly in 3 months and the next significant improvement was presented in the 12-month reevaluation. For example, from full flexion to the full extension (FE) the ROM of lumbar curve was 7.31° ± 1.75°, almost tripled in 3 months (20.64° ± 2.74°) and quadrupled (28.9° ± 2.4°) in the final assessment. Also, the ROM of thoracic curvature from the upright position to the full extension (AΕ) from 5.72° ± 1.76° in 15 days increased to 18.33° ± 1.83° in 12 months.

#### 3.2.2. Frontal Plane

There was no statistically significant change in any parameter in 6 months in comparison with 3-month reevaluation. Most of the improvements were recorded after 6 months. For example, lumbar curvature from the standing position to the full left lateral bending (SL) increased at 7.74° ± 0.68° at six months postoperatively compared with 15-day evaluation (3.43° ± 0.76°). Primarily, statistically significant changes existed only in the comparison with the evaluation in 15 days. In the assessment of ROM of lumbar curvature from standing position to the full right lateral bending (SR), an increase to 11.37° ± 1.15° was recorded in 12 months, in comparison with the mean value which was presented in 15 days (4.15° ± 0.83°). Similarly, the ROM of the thoracic curvature from the full left lateral bending to the full right lateral bending (LR), from 35.56° ± 2.47° in 15 days, almost doubled in the final assessment (60.8° ± 3.02°).

All the parameters which showed statistically significant changes between reevaluations are presented in Table 3.

### 3.3. Questionnaires

#### 3.3.1. ODI

There were statistically significant improvements between all the reevaluations and significant reduction of the score.

#### 3.3.2. VAS

Between all reevaluations a statistically significant decrease was recorded up to 6 months, while the assessment at 12 months did not exhibit any statistically significant change.

The mean values and the statistical significant changes for the questionnaires are presented in Table 4.

### 3.4. Comparison of the CG with the TG

The main statistically significant changes between the two groups are shown in Table 5. In all the reevaluations TG was inferior to CG, except for the measurement of ROM of Sac_Hip in the positions AF, AE, and FE where it was significantly superior.

### 3.5. Comparison of BKP with BKP plus Short Minimal Invasive Fusion

Both in the sagittal and in the frontal plane, for all the parameters, no statistically significant differences were recorded.

### 3.6. Comparison of Osteoporotic Fracture with Traumatic Fracture

According to the type of fracture, in the final evaluation, patients with osteoporotic fracture showed statistically significant lower mean value (*p* = 0.034, 95% CI 1.549, 10.092) only in the lumbar curve in full flexion. Also, patients with osteoporotic fractures presented lower values in the measurement of Incl in the frontal plane both in left lateral bending (*p* = 0.045, 95% CI 18.624, 25.514) and in upright position (*p* = 0.022, 95% CI 0.903, 2.348). Finally, patients with traumatic fracture showed lower mean value (*p* = 0.002, 95% CI 8.22%, 15.06%) in ODI, which means that patients with osteoporotic fractures have higher functional disability degree.

## 4. Discussion

It is well accepted that disturbances in the curvatures and functional limitations of the spinal column following a fracture induce significant problems, especially in the elderly [19]. For this purpose, the spine must be examined as a whole along with the spinopelvic angulation, without the evaluation process being aggravating for the participant or having radiation exposure, especially for repeated evaluations [11]. In the present study a noninvasive device was used, which requires short time for assessment and evaluates the spine from C7 to S2-S3.

### 4.1. Fractures

In our study most of the fractures occurred in T12 and L1 vertebrae. In total, 71.1% of all fractures were located in the thoracolumbar spine (TLS) junction (T11-L2). This is supported by the literature, where it is mentioned that over 60% of VFs occur in the TLS junction [9].

### 4.2. Spine Curvatures

#### 4.2.1. Sagittal Plane

In a randomized trial, BKP was compared to nonsurgical treatment. Early positive results of BKP, clinically, radiologically, and in QOL, were shown at the first month [7]. Another study presented improvements up to 24 months [20]. However, most of the studies are estimating the height of the vertebrae, back pain, and QOL and not the curvatures and the mobility of the spine.

In our study a significant element is the fact that most of the improvements were presented early from the 3-month evaluation and in some parameters those improvements continued up to 12 months. Typical examples are the measurements of Sac_Hip and Incl. It is well known that Sac_Hip angle is directly correlated with spine curvatures and that spine deformity and imbalance in the sagittal plane create compensatory mechanisms on the spinopelvic complex. Also, Sac_Hip angle changes with age, rotating backward [21–23]. Therefore, significant improvement of this parameter demonstrates the total decrease of deformities and imbalance.

Regarding the lumbar curve decreased lordosis, which was recorded in the 15-day evaluation, might be due to the presence of paraspinal muscle spasm [24] and age of the patients, since it is known that lumbar lordosis tends to decrease with age [25]. Generally, curvatures of 20°–60° have been recorded in people with osteoporosis or VF [8]. Also, hypolordosis was recorded in another study where people with osteoporosis were examined with the method of Spinal Mouse [26]. In this particular study it is emphasized that decreased lordosis increases the possibility for a fall and therefore for a new fracture due to induced anteroposterior imbalance and posterior pelvic tilt. From the above, it is clear that the improvement in the lumbar curve in our study, which was shown early from the 3 months, has a great importance and acts positively in many ways.

#### 4.2.2. Frontal Plane

In the present study all the parameters showed improvement mainly after 6 and 12 months suggesting that, in comparison with the sagittal plane, these improvements appear at lower rate.

Generally, even though the positive results of BKP in the curvatures of the spine are shown early many factors tend to improve up to 12 months.

### 4.3. Spinal Mobility

#### 4.3.1. Sagittal Plane

It has been proven that reduced spinal mobility causes significant impairment, especially in the elderly [8]. In addition, there is a proportional correlation between decreased mobility and QOL in older patients and in patients with osteoporosis [27]. In the present study significant improvement was recorded regarding mobility of all curvatures (thoracic, lumbar, and Sac_Hip) and total mobility of the trunk (Incl) as early as 3 months postoperatively. Surgical treatment has tripled in many cases the mobility, which remained unchanged between 3 and 6 months, and then showed an additional improvement in the 12-month evaluation. The latter is probably related to increased risk for adjacent VF. This is in accordance with others who found that an adjacent fracture often occurs one year postoperatively [28]. In general, the significant increase of mobility induces a noteworthy gain and improves the QOL while it simultaneously reduces all the aforementioned dangers.

#### 4.3.2. Frontal Plane

Similarly with the results that were recorded for spinal curvatures, mobility improvements in the frontal plane were demonstrated mainly 6 months postoperatively. To the best of our knowledge, there are not any studies which examined the mobility of the spine in the frontal plane. There is no obvious explanation why these improvements, in that particular plane, presented later than in sagittal plane. One hypothesis for this could be that spinal deviations in frontal plane are correlated with alteration of loading which is applied to the facet joints [29]. These joints are characterized by limited mobility.

### 4.4. Questionnaires

Regarding VAS score, it is known that BKP and vertebroplasty offer instant and significant relief from pain and present better results in comparison with conservative treatment [5, 10]. In our study, although pain was significantly reduced postoperatively, yet it was higher in comparison with other studies which used BKP plus fixation [9] or BKP alone [20]. However, there are studies where postoperative level of pain was approximately the same with our study [7, 30]. The reasons for these differences might be the different management and the methodology of each study, the bias that arise from the evaluation of feeling of pain (if participants answered regarding the maximum feeling of pain or the average pain that they felt), the case that some participants might have taken analgesics, and other parameters [10]. It must be noted that in the present study the question was about the maximum feeling of pain. Generally, pain improvement was significant, especially in the 3- and 6-month reevaluations. Most of the studies record values from 0 to 3 [7, 9, 10, 20, 30]. After 6 months pain levels do not show further improvements. Probably, similar results from 6 to 12 months might be due to the fact that some of the patients got better and some others got worse, creating a balance.

Also, ODI evaluation showed significant improvement of functionality. The superiority of kyphoplasty over the other methods and the gradual reduction of score throughout the first year has been recorded in the literature [10, 20], a fact that was also recorded in our study.

### 4.5. Comparison of the CG with the TG and BKP with BKP plus Short Minimal Invasive Fusion

Although there were very good results during all reevaluations, regarding spinal curves, mobility, pain, and functionality, finally the TG was more inferior than CG, especially in the parameters of lumbar spine. On the other hand, TG showed better mobility in Sac_Hip than CG. These results might have compensatory action as Sac_Hip angle and mobility are correlated directly with lumbar lordosis and mobility [8, 11].

Finally, in the present study no differences were recorded between the two treatment methods. One particular study showed differences only in VAS, ODI, and kyphosis, which was evaluated radiologically on the basis of Cobb angle, showing that internal fixation with percutaneous kyphoplasty was inferior to kyphoplasty alone. However, the bias of the above study was that the participants had an increased average of age (all > 65) and only burst fractures were evaluated [31].

### 4.6. Comparison of Osteoporotic Fracture with Traumatic Fracture

Patients with osteoporotic fractures had poorer results in comparison with traumatic fractures. The main reason for the above is that osteoporotic patients are elderly with functional impairments, reduced bone quality, and muscular weakness. Even though it has been proven that in people over 50 most of the fractures are due to osteoporosis, compared to other parameters such as trauma, metastasis, and multiple myeloma [32], the influence of the nature of the fracture in the final result, in accordance with all the associated factors, needs further investigation.

## 5. Conclusion

The present study is the first that examines the entire spine, regarding both spinal curves and mobility, after surgical treatment of a fracture. In addition, this study evaluates the whole spine in two planes and compares all the parameters giving a comprehensive and complete picture of the postoperative patient's status.

Both BKP and BKP plus fixation show significant early improvements regarding structure and mobility of the spine, especially in lumbar spine and Sac_Hip, which improve posture, balance, and QOL. At the same time they reduce deformities and limit the risk for a subsequent fall-related injury. In most of the parameters, there is a constant progress during reevaluations. Moreover, pain and disability reduce significantly and, combined with improvements in structure of the spine, cumulatively produce a clinically positive effect.

## Figures and Tables

**Table 1:** Demographic and anthropometric characteristics of the participants.

	Treatment Group (TG) *n* = 39	Control Group (CG) *n* = 39
Gender		
Male	*n* = 19 (48.7%)	*n* = 17 (43.6%)
Female	*n* = 20 (51.3%)	*n* = 22 (56.4%)
Age	57.15 (±15.97)	51.82 (±11.74)
Height	1.66 (±0.08)	1.69 (±0.08)
Weight	74.26 (±10.67)	73.03 (±13.18)
BMI	26.97 (±3.58)	25.62 (±3.55)

**Table 2:** Spine curvatures measurements for all positions in sagittal and frontal plane.

	Spinal curvatures					
	3 versus 15	6 versus 15	12 versus 15	6 versus 3	12 versus 3	12 versus 6
Sagittal plane						
Upright position						
Sac_Hip	*p* < 0.001 (2.650, 8.171)	*p* = 0.006 (6.986, 8.143)	*p* = 0.001 (1.458, 7.670)			
Lumbar curve	*p* = 0.004 (1.484, 10.157)	*p* = 0.045 (0.080, 10.176)	*p* = 0.006 (1.318, 10.785)			
Full flexion						
Sac_Hip	*p* < 0.001 (19.884, 37.244)	*p* < 0.001 (26.909, 47.604)	*p* < 0.001 (34.533, 55.364)	*p* = 0.026 (0.694, 16.691)	*p* < 0.001 (7.962, 24.807)	*p* < 0.001 (2.961, 12.424)
Lumbar curve	*p* = 0.047 (0.062, 14.246)	*p* < 0.001 (5.360, 19.101)	*p* < 0.001 (8.632, 22.958)		*p* = 0.002 (2.679, 14.603)	*p* = 0.012 (0.566, 6.562)
Incl	*p* < 0.001 (24.067, 47.472)	*p* < 0.001 (37.423, 60.116)	*p* < 0.001 (47.613, 70.695)	*p* = 0.006 (2.911, 23.089)	*p* < 0.001 (12.627, 34.142)	*p* < 0.001 (4.593, 16.177)
Full extension						
Sac_Hip			*p* = 0.021 (0.683, 11.933)			
Thoracic curve			*p* = 0.001 (3.293, 17.271)			
Lumbar curve	*p* = 0.012 (1.011, 11.348)	*p* = 0.037 (0.258, 12.357)				
Incl	*p* < 0.001 (6.204, 13.591)	*p* < 0.001 (9.711, 17.519)	*p* < 0.001 (11.853, 20.404)		*p* = 0.049 (0.014, 7.422)	*p* = 0.001 (2.263, 10.198)
Frontal plane						
Upright position						
Sac_Hip		*p* = 0.018 (0.631, 2.236)	*p* = 0.003 (0.586, 3.660)		*p* = 0.045 (0.021, 2.620)	
Left lateral bending						
Sac_Hip	*p* = 0.009 (0.493, 4.815)	*p* < 0.001 (2.331, 6.740)	*p* < 0.001 (2.534, 8.153)		*p* = 0.023 (0.254, 5.125)	
Thoracic curve	*p* < 0.001 (6.342, 15.852)	*p* < 0.001 (8.571, 20.352)	*p* < 0.001 (10.744, 20.984)			
Lumbar curve			*p* = 0.015 (0.448, 6.040)			
Incl	*p* < 0.001 (3.384, 10.129)	*p* < 0.001 (6.000, 13.338)	*p* < 0.001 (8.024, 15.458)		*p* = 0.001 (1.559, 8.410)	*p* = 0.039 (0.071, 4.073)
Right lateral bending						
Thoracic curve	*p* = 0.010 (1.182, 12.243)	*p* < 0.001 (4.855, 15.530)	*p* = 0.001 (3.444, 15.320)			
Lumbar curve	*p* < 0.001 (2.398, 8.402)	*p* < 0.001 (5.082, 10.682)	*p* < 0.001 (5.638, 12.300)	*p* = 0.035 (0.114, 4.850)	*p* = 0.002 (1.078, 6.061)	
Incl	*p* < 0.001 (3.048, 9.516)	*p* < 0.001 (4.525, 10.485)	*p* < 0.001 (5.205, 12.795)			

*p* value and CI 95% (Evaluation 3 versus 15: 3 months versus 15 days, 6 versus 15: 6 months versus 15 days, 12 versus 15: 12 months versus 15 days, 6 versus 3: 6 months versus 3 months, 12 versus 3: 12 months versus 3 months, 12 versus 6: 12 months versus 6 months).

**Table 3:** Statistically significant changes in the mobility of the spine in the sagittal and frontal plane among reevaluations.

	Spinal mobility					
	3 versus 15	6 versus 15	12 versus 15	6 versus 3	12 versus 3	12 versus 6
Sagittal plane						
AF						
Sac_Hip	*p* < 0.001 (14.799, 31.611)	*p* < 0.001 (23.021, 42.159)	*p* < 0.001 (30.196, 50.625)	*p* = 0.02 (1.066, 17.703)	*p* < 0.001 (8.491, 25.920)	*p* = 0.002 (2.201, 13.449)
Lumbar curve	*p* < 0.001 (5.152, 20.848)	*p* < 0.001 (10.427, 24.445)	*p* < 0.001 (14.916, 28.673)		*p* = 0.014 (1.299, 16.290)	*p* = 0.045 (0.066, 8.652)
Incl	*p* < 0.001 (23.693, 47.999)	*p* < 0.001 (37.717, 60.847)	*p* < 0.001 (48.134, 71.763)	*p* = 0.009 (2.567, 24.305)	*p* < 0.001 (12.690, 35.515)	*p* < 0.001 (4.251, 17.082)
AE						
Sac_Hip	*p* < 0.001 (3.251, 11.056)	*p* < 0.001 (4.388, 14.074)	*p* < 0.001 (5.721, 16.074)			
Thoracic curve	*p* = 0.048 (0.032, 12.532)		*p* < 0.001 (5.577, 19.654)		*p* = 0.03 (0.424, 12.242)	*p* = 0.004 (1.623, 11.813)
Incl	*p* < 0.001 (6.382, 13.208)	*p* < 0.001 (9.138, 16.913)	*p* < 0.001 (11.200, 19.416)		*p* = 0.003 (1.496, 9.529)	
FE						
Sac_Hip	*p* < 0.001 (21.626, 39.143)	*p* < 0.001 (30.750, 52.994)	*p* < 0.001 (39.799, 69.714)	*p* = 0.024 (1.047, 21.927)	*p* < 0.001 (9.736, 32.007)	*p* < 0.001 (4.311, 14.458)
Thoracic curve	*p* = 0.034 (0.386, 14.486)		*p* < 0.001 (5.390, 21.072)		*p* = 0.039 (0.203, 11.386)	*p* = 0.026 (0.546, 12.377)
Lumbar curve	*p* = 0.001 (4.732, 21.934)	*p* < 0.001 (11.454, 26.034)	*p* < 0.001 (14.042, 29.128)		*p* = 0.033 (0.465, 16.048)	
Frontal plane						
SL						
Sac_Hip		*p* = 0.02 (0.297, 4.914)			*p* = 0.032 (0.193, 6.248)	
Thoracic curve	*p* < 0.001 (6.482, 15.800)	*p* < 0.001 (8.196, 19.276)	*p* < 0.001 (10.221, 20.666)			
Lumbar curve		*p* < 0.001 (0.026, 4.867)	*p* < 0.001 (2.068, 7.907)			
Incl	*p* < 0.001 (3.067, 9.472)	*p* < 0.001 (5.573, 12.247)	*p* < 0.001 (7.277, 14.502)		*p* = 0.002 (1.316, 7.925)	
SR						
Thoracic curve	*p* = 0.004 (1.713, 11.625)	*p* < 0.001 (5.342, 16.494)	*p* < 0.001 (3.691, 15.914)			
Lumbar curve	*p* = 0.002 (1.505, 8.177)	*p* < 0.001 (2.433, 9.936)	*p* < 0.001 (3.147, 11.304)			
Trunk Incl	*p* < 0.001 (3.214, 10.325)	*p* < 0.001 (15.043, 11.485)	*p* < 0.001 (5.900, 13.803)			
LR						
Sac_Hip		*p* = 0.031 (0.209, 6.473)	*p* = 0.013 (0.555, 8.804)			
Thoracic curve	*p* < 0.001 (9.836, 25.784)	*p* < 0.001 (16.901, 32.407)	*p* < 0.001 (16.964, 33.526)			
Lumbar curve	*p* < 0.001 (3.642, 10.881)	*p* < 0.001 (6.106, 14.873)	*p* < 0.001 (7.407, 17.019)		*p* = 0.003 (1.379, 8.524)	
Incl	*p* < 0.001 (7.096, 18.981)	*p* < 0.001 (11.021, 23.328)	*p* < 0.001 (13.575, 27.907)		*p* = 0.04 (1.860, 13.545)	

*p* value and CI 95% (Evaluation 3 versus 15: 3 months versus 15 days, 6 versus 15: 6 months versus 15 days, 12 versus 15: 12 months versus 15 days, 6 versus 3: 6 months versus 3 months, 12 versus 3: 12 months versus 3 months, 12 versus 6: 12 months versus 6 months).

**Table 4:** Statistically significant improvements from the evaluation of the questionnaires ODI and VAS.

	ODI 95% CI	VAS-back 95% CI
Mean value and SD		
15 days	69.36% ± 1.45%	5.69 ± 0.18
3 months	45.51% ± 1.97%	3.59 ± 0.17
6 months	17.56% ± 1.65%	1.62 ± 0.17
12 months	11.64% ± 1.69%	1.28 ± 0.28
Comparison of reevaluations		
3 months versus 15 days	*p* < 0.001 (21.091, 28.601)	*p* < 0.001 (1.783, 2.423)
6 months versus 15 days	*p* < 0.001 (47.386, 56.204)	*p* < 0.001 (3.542, 4.612)
12 months versus 15 days	*p* < 0.001 (53.500, 61.936)	*p* < 0.001 (3.704, 5.117)
6 months versus 3 months	*p* < 0.001 (22.633, 31.264)	*p* < 0.001 (1.419, 2.530)
12 months versus 3 months	*p* < 0.001 (28.811, 36.933)	*p* < 0.001 (1.576, 3.039)
12 months versus 6 months	*p* = 0.005 (1.359, 10.487)	

**Table 5:** Statistically significant differences between the CG and TG, based on the measurements of Spinal Mouse (*p* value and CI 95%).

	TG versus CG			
	Sac_Hip	Lumbar curve	Thoracic curve	Incl
Sagittal plane				
Upright position	*p* < 0.001 (12.229, 16.950)	*p* < 0.001 (26.426, 32.394)		
Full flexion		*p* = 0.001 (8.179, 16.077)		
Full extension	*p* < 0.001 (0.009, 6.778)	*p* < 0.001 (29.364, 36.79)		
AF	**p** < 0.001 **(47.359, 54.974)**	*p* < 0.001 (37.221, 45.728)		
AE	**p** = 0.002 **(9.038, 13.372)**	*p* < 0.001 (1.599, 5.786)	*p* = 0.015 (12.712, 17.8)	
FE	**p** < 0.001 **(57.552, 67.318)**	*p* < 0.001 (39.682, 50.497)		
Frontal plane				
Upright position	*p* < 0.001 (0.293, 1.689)	*p* = 0.001 (2.945, 4.575)	*p* = 0.028 (4.731, 7.064)	*p* = 0.003 (0.385, 1.384)
Left bending		*p* < 0.001 (12.718, 15.69)		
Right bending	*p* = 0.020 (4.805, 6.723)	*p* = 0.001 (10.11, 13.78)		*p* < 0.001 (20.739, 24.512)
SL		*p* = 0.002 (9.102, 11.786)		*p* = 0.008 (19.963, 23.368)
SR		*p* < 0.001 (13.646, 17.765)		*p* < 0.001 (12.023, 17.783)
LR		*p* = 0.005 (12.019, 18.543)	*p* < 0.001 (32.62, 47.208)	*p* < 0.001 (23.263, 34.014)
